# Work Engagement among Rescue Workers: Psychometric Properties of the Portuguese UWES

**DOI:** 10.3389/fpsyg.2017.02229

**Published:** 2018-01-22

**Authors:** Jorge Sinval, Alexandra Marques-Pinto, Cristina Queirós, João Marôco

**Affiliations:** ^1^Faculty of Philosophy, Sciences and Letters of Ribeirão Preto, University of São Paulo, São Paulo, Brazil; ^2^Faculty of Psychology and Education Sciences, University of Porto, Porto, Portugal; ^3^Faculdade de Psicologia, Centro de Investigação em Ciência Psicológica, Universidade de Lisboa, Lisboa, Portugal; ^4^William James Centre for Research, ISPA-Instituto Universitário, Lisboa, Portugal

**Keywords:** work engagement, rescue workers, UWES, reliability, validity evidence, measurement invariance

## Abstract

Rescue workers have a stressful and risky occupation where being engaged is crucial to face physical and emotional risks in order to help other persons. This study aims to estimate work engagement levels of rescue workers (namely comparing nurses, firefighters, and police officers) and to assess the validity evidence related to the internal structure of the Portuguese versions of the UWES-17 and UWES-9, namely, dimensionality, measurement invariance between occupational groups, and reliability of the scores. To evaluate the dimensionality, we compared the fit of the three-factor model with the fit of a second-order model. A Portuguese version of the instrument was applied to a convenience sample of 3,887 rescue workers (50% nurses, 39% firefighters, and 11% police officers). Work engagement levels were moderate to high, with firefighters being the highest and nurses being the lowest engaged. Psychometric properties were evaluated in the three-factor original structure revealing acceptable fit to the data in the UWES-17, although the UWES-9 had better psychometric properties. Given the observed statistically significant correlations between the three original factors, we proposed a 2^nd^ hierarchal structure that we named work engagement. The UWES-9 first-order model obtained full uniqueness measurement invariance, and the second-order model obtained partial (metric) second-order invariance.

## Introduction

Rescue workers' main tasks are to bring people out of danger after accidents, attacks, disasters, etc., are the first on the scene of an emergency or provide care to victims who are suffering. Firefighters, police officers or health professionals working in urgency or pre-hospital departments can be considered as rescue workers, having a highly risky and stressful occupation (Stassen et al., 2013). However, they are very motivated and engaged with their tasks frequently facing physical and emotional risks to help other persons (Shakespeare-Finch, 2011). To better understand work engagement among rescue workers, the theoretical framework is presented beginning with the work engagement concept and its study among rescue workers, followed by some considerations about work engagement measurement using the Utrecht Work Engagement Scale (UWES).

### Work engagement concept and its study among rescue workers

Considering the frequent occurrence of disasters due to natural or human causes, particularly attention must be given to rescue workers dealing with critical incidents or disasters, since chronic emotional demands and coping with stressful situations harms their health and the services provided by their institution (Queirós et al., 2013; Rabjohn, 2013; International Labour Organization, 2016). Therefore, these professionals can develop negative emotional states, psychological disorders or mental diseases (e.g., anxiety, loneliness, panic attacks, depression, post-traumatic stress disorder, vicarious trauma), but particularly, they can experience chronic job stress and become vulnerable to burnout (Cieslak et al., 2014; Katsavouni et al., 2016; Krok, 2016). Several studies have emphasized the relationships between burnout and work engagement (Schaufeli et al., 2002a; Maslach and Leiter, 2008; Leon et al., 2015; Leiter and Maslach, 2017; Schaufeli and De Witte, 2017), suggesting that promoting work engagement can prevent burnout (Argentero and Setti, 2011), thus ameliorating rescue workers' mental health and helping their stress management (Nishi et al., 2016; O'Neill and Rothbard, 2017). In fact, work engagement seems to be an important psychological resource for rescue workers, since it may protect them from the risk of developing negative health effects (Setti and Argentero, 2014), depression, sleep disturbances, relational conflicts, burnout, compassion fatigue or vicarious trauma (Lenthall et al., 2009; Kumar, 2011; Ray et al., 2013), or presenteeism (Burton et al., 2017).

For several decades, Psychology has focused on pathology and psychological illness, which has led to a restricted view of human behavior that has consequently contributed to the depreciation of resources, qualities and skills of human beings as promoters of individual growth and psychological well-being (Diener et al., 1999). However, the recent emergence of Positive Psychology has counteracted this focus, valorizing the study of the positive part of human experience (Luthans, 2002), aiming to increase the quality of life, to promote individual qualities, resources, and characteristics that increase psychological well-being and growth (Seligman and Csikszentmihalyi, 2000). This approach within Positive Psychology explores building positive qualities, instead of focusing on repairing psychological illness. One of the theoretical concepts that arises within this perspective is work engagement (Schaufeli et al., 2002a), focused on the positive aspects of behaviors, the well-being and the optimal functioning of each person (Schaufeli and Salanova, 2007).

Although work engagement is a relatively new construct, it has been viewed as being comprised of slightly different aspects, such as physical, cognitive, emotional and mental expression by employees within the exercise of their roles (Kahn, 1990). Distinctions between organizational and job engagement have appeared regarding employees' different roles suggesting that engaged workers invest and dedicate themselves deeply into their tasks, which are viewed as motivated situations (Saks, 2006). Other authors (Maslach and Leiter, 1997, 2017; Maslach et al., 2001) have considered work engagement as a concept diametrically opposed to burnout, being defined as an energetic state of involvement in vital personal activities, but quite different from job satisfaction or organizational commitment (Hallberg and Schaufeli, 2006).

While the discussion about burnout and work engagement as different constructs or as opposite poles of the same construct continues (Schaufeli et al., 2002b; Macey et al., 2009; Cole et al., 2012; Leon et al., 2015; Leiter and Maslach, 2017; Schaufeli and De Witte, 2017), we adopt Schaufeli and colleagues' perspective on work engagement as a positive motivational, cognitive-affective, and emotional persistent state (Schaufeli and Salanova, 2007; Schaufeli, 2017b), divided into three factors or dimensions: vigor, dedication and absorption. Vigor is related to persistence, energy and mental resilience during work, while dedication implies being involved in the work and living with enthusiasm, inspiration, and pride, and finally, absorption means to be concentrated and immersed in one's work, with the perception that time flies (Schaufeli et al., 2002b). Thus, workers with high levels of work engagement see themselves as full of energy when facing their professional activities (Albrecht, 2010).

Work engagement is also related with other important occupational health and organizational outcomes, including in-role and extra-role behavior (Bakker et al., 2004). It is positively correlated with performance (Cooper and Quick, 2017; Reijseger et al., 2017); more specifically, a direct and positive relation has been found with job performance and a direct and negative relation with turnover intention (Karatepe and Avci, 2017; Kim, 2017), proactive behaviors and employee intrapreneurship (Gawke et al., 2017), job crafting and personal resources (Xanthopoulou et al., 2009; Vogt et al., 2016), general job resources (Schaufeli et al., 2009), structural and social resources (Harju et al., 2016), and job, career and life satisfaction (Karatepe and Karadas, 2015).

### Work engagement measurement with the utrecht work engagement scale

In line with its three-dimensional definition, the work engagement measure was divided into three sub-scales, in a psychometric instrument called Utrecht Work Engagement Scale (UWES), a self-report instrument that includes the vigor, dedication and absorption factors (with six, five and six items respectively). Although the UWES presents good psychometric properties. it is common to find very strong correlations between the factors (Chughtai and Buckley, 2013; Agarwal, 2014), leading some studies to prefer the single factor measure (Shimazu et al., 2008; Seppälä et al., 2009; Klassen et al., 2012), while others (Shimazu et al., 2008; Alok, 2013) tested UWES as a second-order construct (Edwards, 2001). Kulikowski (2017) conducted a literature review with 21 studies using Confirmatory Factor Analysis (CFA) of UWES; six of these recognized the three-factor version as superior to the one-factor solution, six studies found the opposite, eight studies found both structures as equivalent, and one study failed to confirm both structures. However, usually, the CFAs present good results with the three-factor structure. Moreover, various studies found invariance between different occupational and racial groups and countries (Storm and Rothmann, 2003; Xanthopoulou et al., 2012), and relative longitudinal invariance for the short version (Seppälä et al., 2009). This shorter version with nine items is divided into the same three dimensions, with three items per dimension (Schaufeli et al., 2006).

The UWES has been used in several studies with rescue workers and translated into many languages (Schaufeli and Bakker, 2003). Several studies have shown that the structure of the UWES remains unchanged among different occupational groups. Table 1 presents a synthesis of 30 studies between 2003 and 2017, conducted mainly in Europe but also in America, Asia, Africa and Oceania, with professionals with occupation statuses similar to the ones of our study, revealing that work engagement levels are moderate to high among these rescue workers.

**Table 1:** UWES research among rescue workers.

**Authors**	**Continent**	**Country**	**Occupational group**	***N***	**Results**	**Work Engagement findings**
Vallières et al., 2017	Africa	Sierra Leone	Community health workers	334	No differences between genders, literate and illiterate respondents; some differences among educational levels.	There was support for the utilization of the shortened UWES-9.
Naudé and Rothmann, 2015	Africa	South Africa	Emergency Medical Technicians	318	A two-factor model was found.	There was construct equivalence of work engagement for white and black people.
Storm and Rothmann, 2003	Africa	South Africa	Police Officers	2,396	There is a 3-factor model of work engagement.	UWES can be used to compare work engagement of different race groups.
Siller et al., 2016	America	United States of America	Nurses	43	Means: Work Engagement = 4.40; Vigor = 4.30; Dedication = 4.80; Absorption = 4.20.	The perceptions of shared governance in emergency nurses were associated with work engagement.
Hu et al., 2017	Asia	China	Nurses	172	Means: Nurses: Vigor = 2.13–2.51, Dedication = 2.02–2.24, Absorption = 2.01–2.27 Police officers: Vigor = 3.03–3.14, Dedication = 2.93–2.97, Absorption = 2.85–2.88.	Participants who experienced job demands-resources reported a significant increase in burnout and a significant decrease in work engagement.
			Police Officers	273		
Fong and Ho, 2015	Asia	China	Health	1,112	None of the three Maximum Likelihood-based models showed an adequate fit to the data.	The Bayesian structural equation modeling supported the overall factor as an adequate and parsimonious representation of work engagement.
Shimazu et al., 2008	Asia	Japan	Engineers	794	One-factor structure was invariant across the samples	This version provided reliability (internal consistency and stability), factorial invariance, and construct validity evidence.
			Nurses	1,540		
Panthee et al., 2014	Asia	Nepal	Nurses	438	Means: Work engagement: 18–30 years = 4.81; 31–45 years = 4.75; 46–59 years = 5.36	This version had satisfactory psychometric properties and provided supportive evidence.
Aboshaiqah et al., 2016	Asia	Saudi Arabia	Nurses	980	Means: Vigor = 4.00; Dedication = 4.60; Absorption = 3.90	There were high levels of work engagement among the nurses working in hospitals in different health sectors.
Van Bogaert et al., 2017	Europe	Belgium	Nurses	751	Vigor explained 20% of the variance in job outcomes. Absorption had ≤ 5% of the relevant direct impact on quality of care.	Nurses' work engagement and work characteristics mediated the effect of practice environment on quality of care and job outcomes.
Seppälä et al., 2009	Europe	Finland	Multi-occupational	9,404	Means: Vigor = 4.51, Dedication = 4.82, Absorption = 3.82.	Work engagement showed evidence of being a highly stable indicator of occupational well-being.
			(Health care)	(736)		
Gillet et al., 2013	Europe	France	Police Officers	235	Means: Vigor = 3.77–3.92; Dedication = 3.76–4.10; Absorption = 3.85–4.01	Promotion of self-determined motivation can improve police officers' work engagement.
				147		
Tomietto et al., 2016	Europe	Italy	Nurses	519	Means: Work engagement: Medical	Nursing teams' work engagement was an important and effective factor
			Nursing students	519	wards = 5.4; Surgical wards = 5.5; Rehabilitation services = 5.7; Critical wards = 5.6; Paediatric wards = 5.3 (scale from 1 to 7).	to improve nursing students' learning experience within a clinical context.
van Gelderen and Bik, 2016	Europe	Netherlands	Police officers	114	Mean: Work engagement = 4.92	Supervisor support mediated the positive relationship between commitment and work engagement/extra-role performance.
Bolier et al., 2014	Europe	Netherlands	Health care	366	Means: Work engagement online group = 4.36–4.46 Work engagement control group = 4.21–4.37	The workers' health surveillance (WHS) module, including screening, feedback and offer of online interventions had a small positive effect on work engagement.
Breevaart et al., 2012	Europe	Netherlands	Multi-occupational	271	The three-factor multilevel model had better fit to the data.	UWES can be used to measure both trait and state work engagement.
			(Health)	(Not available)		
Nerstad et al., 2010	Europe	Norway	Nurses	109	Factorial invariance and the internal consistencies were acceptable.	Norwegian short version was recommended over the UWES-17.
			Police officers	150		
			Multi-occupational	1,266		
Richardsen et al., 2006	Europe	Norwegian	Police officers	150	Mean: Work engagement = 4.16	Work engagement partially mediated the effects of individual characteristics, job demands and job resources on organizational commitment and self-efficacy.
Ângelo and Chambel, 2015	Europe	Portugal	Firefighters	651	Means: Vigor = 4.86–4.97; Dedication = 5.29–5.37	There was no causal effect of supervisory support on work engagement, which highlights the need for a customized intervention that focuses on the specific reality of rescue mission firefighters.
Montero-Marin et al., 2016	Europe	Spain	Health care	440	Vigor, Dedication, and Absorption associated with burnout subtypes.	There was a relation between work engagement and burnout subtypes: directly related with frenetic subtype, and inversely related with underchallenged and worn-out subtypes.
Spontón et al., 2012	America	Argentina	Multi-occupational	337	No statistically significant difference between occupations.	Both two-factor and three-factor models were plausible.
			(Health)	(Not available)		
Vazquez et al., 2015	America	Brazil	Health	113	Mean: Health professionals Work engagement = 4.2	Health professionals presented lower work engagement levels than other occupational groups.
			(Various)	(1,167)		
Espinoza-Parra et al., 2015	America	Chile	Police officers	985	Mean: Work engagement = 3.68	Work engagement and group identification mediated the effect of transformational leadership in job satisfaction.
Gilchrist et al., 2013	America	Chile	Health	165	A two-factor model was found (UWES-17): involvement with work and enthusiasm for work.	The proposed version had appropriate psychometric properties.
Hernandez-Vargas et al., 2016	America	México	Health	475	UWES-9 is preferable when compared with the UWES-15.	There was validity evidence to use the UWES-9 with Mexican health professionals.
Brunetto et al., 2012	Oceania	Australia	Police officers	193	Mean: Work engagement = 4.32 (scale from 1 to 6).	Organizational commitment was found to partially mediate the causal relationship between employee work engagement and turnover intentions.
Tuckey et al., 2012	Oceania	Australia	Firefighters	540	Mean: Work engagement = 3.81	Increased levels of cognitive demands and cognitive resources partially mediated the relationship between empowering leadership and work engagement.
Poulsen et al., 2011	Oceania	Australia	Health (cancer workers)	579	Overall, 34.5% of the cancer workers were highly engaged in their work.	There was a positive association between work engagement and non-shift workers.
Schaufeli et al., 2006	Various	Australia, Belgium, Canada, Finland, France, Germany, Netherlands, Norway, South Africa, Spain	Multi-occupational	14,521	Means: Police officers: Vigor = 4.14; Dedication = 4.55; Absorption = 4.05 Health care workers: Vigor = 3.94; Dedication lower than police officers; Absorption = 3.55.	Work engagement can be conceived as the opposite of burnout. UWES-9 showed acceptable psychometric properties.
			Health care	2,777		
			Police officers	2,650		
Thian et al., 2013	Various	Various	Nurses	254	Positive affectivity had a significant positive relationship with work engagement.	Work engagement and positive affectivity were related and can be enhanced (together with stress reduction) through worksite interventions/strategies.
				412		
				167		

Considering previous studies with UWES among these professionals, our research question was to investigate if UWES is an adequate instrument to assess rescue workers' engagement. Thus, this study aims to estimate work engagement levels of rescue workers (namely comparing nurses, firefighters, and police officers) and to assess the validity evidence related with the internal structure of the Portuguese versions of the UWES-17 and UWES-9, namely to evaluate their dimensionality (comparing the fit of the three-factor first-order model of the UWES-17 and UWES-9, and of a possible UWES-9 second-order model), measurement invariance between the three occupational groups, and reliability of the scores, according to the *Standards for Educational and Psychological Testing* framework (American Educational Research Association et al., 2014). According to these aims, we have as hypotheses: (H1) UWES-17 presents validity evidence that supports its usage among rescue workers; (H2) UWES-9 presents validity evidence that supports its usage among rescue workers; (H3) UWES-9 presents measurement invariance that allows for comparing different occupational groups; (H4) UWES-9 reveals different work engagement levels between the three different occupational groups of rescue workers.

## Methods

### Participants

A sample of 3,887 Portuguese rescue workers completed the UWES-17 (except 428 workers that only completed the UWES-9), with data collected from several research projects (seven studies) developed at the Psychosocial Rehabilitation Laboratory from FPCEUP, between 2010 and 2016. Data were from different Portuguese districts, but with all occupations contributing to the data during the same year. The average age was 33.35 years old (*SD* = 9.84), 50% male, 44% married (and 50% single), 62% at least had a college degree. Regarding occupations, 50% were nurses, 39% voluntary firefighters (according to the Portuguese firefighters' organization, they can be voluntary or professional firefighters; this study collected data from voluntary firefighters, a larger group than professional firefighters) and 11% police officers.

### Measures

A sociodemographic questionnaire was administered, with items such as age, sex, marital status, school level and occupation. Despite other questions specific to each rescuer occupation, these questions were asked to all groups. To assess work engagement, we used the UWES (Table 2) developed by Schaufeli et al. (2002b) in its Portuguese version (Marques-Pinto and Picado, 2011).

**Table 2:** UWES's items.

**Item**	**Original UWES - 17 (Schaufeli and Bakker**, **2003****)**							**Portuguese version (Marques-Pinto and Picado**, **2011****)**						
Never		Almost never	Rarely	Sometimes	Often	Very often	Always	Nunca	Quase nunca	Às vezes	Regularmente	Frequentemente	Quase sempre	Sempre
	0	1	2	3	4	5	6	0	1	2	3	4	5	6
	Never	A few times a year or less	Once a month or less	A few times a month	Once a week	A few times a week	Every day	Nenhuma vez	Algumas vezes por ano	Uma vez ou menos por mês	Algumas vezes por mês	Uma vez por semana	Algumas vezes por semana	Todos os dias
	**VIGOR**							**VIGOR**						
1	At my work, I feel bursting with energy^*^							No meu trabalho sinto-me cheio de energia^*^						
4	At my job, I feel strong and vigorous^*^							No meu trabalho sinto-me com forļa e energia^*^						
8	When I get up in the morning, I feel like going to work^*^							Quando me levanto de manhã apetece-me ir trabalhar^*^						
12	I can continue working for very long periods at a time							Sou capaz de ficar a trabalhar por períodos de tempo muito longos						
15	At my job, I am very resilient, mentally							Sou uma pessoa com muita resistência mental no meu trabalho						
17	At my work I always persevere, even when things do not go well							No meu trabalho sou sempre perseverante (não desisto), mesmo quando as coisas não estão a correr bem						
	**DEDICATION**							**DEDICAÇÃO**						
2	I find the work that I do full of meaning and purpose							Acho que o meu trabalho tem muito significado e utilidade						
5	I am enthusiastic about my job^*^							Estou entusiasmado com o meu trabalho^*^						
7	My job inspires me^*^							O meu trabalho inspira-me^*^						
10	I am proud on the work that I do^*^							Estou orgulhoso do que faļo neste trabalho^*^						
13	To me, my job is challenging							O meu trabalho é desafiante para mim						
	**ABSORPTION**							**ABSORÇÃO**						
3	Time flies when I'm working							O tempo passa a voar quando estou a trabalhar						
6	When I am working, I forget everything else around me							Quando estou a trabalhar esqueļo tudo o que se passa à minha roda						
9	I feel happy when I am working intensely^*^							Sinto-me feliz quando estou a trabalhar intensamente^*^						
11	I am immersed in my work^*^							Estou imerso no meu trabalho^*^						
14	I get carried away when I'm working^*^							“Deixo-me ir” quando estou a trabalhar^*^						
16	It is difficult to detach myself from my job							É-me difícil desligar-me do meu trabalho						

* *Short version (UWES-9)*.

### Procedures

Participants were invited to voluntarily participate after formal authorization from each organization where they were working at and filled out printed versions of the UWES anonymously. The study was approved by the Ethics Committee of University of Porto, Portugal, and written informed consent from all research participants was obtained.

### Data analysis

To study the dimensionality of the UWES versions, a CFA was conducted to verify if the three-factor structure proposed by the UWES' authors presented an adequate fit for this study's sample. We used as the goodness-of-fit indices the TLI (Tucker Lewis Index), NFI (Normed Fit Index), χ^2/^df (ratio chi-square and degrees of freedom), CFI (comparative fit index) and the RMSEA (root mean square error of approximation). The fit of the model was considered good for χ^2/^df smaller than 5, CFI, NFI and TLI values above 0.95 and RMSEA values below 0.08 (Hoyle, 1995; Boomsma, 2000; McDonald and Ho, 2002; Marôco, 2014; Byrne, 2016).

To analyze convergent validity evidence, the average variance extracted (AVE) was estimated as described in Fornell and Larcker (1981). Values of AVE ≥ 0.5 were considered indicative of the constructs' convergent validity evidence, for both versions of the UWES per the proposal by Hair et al. (2009).

Discriminant validity indicates that the items that represent a dimension are not strongly correlated with other dimensions (Fornell and Larcker, 1981; Marôco, 2014). For two factors *x* and *y*, if AVE*x* and AVE*y* ≥ ρ^2^ *xy* (squared correlation between the factors *x* and *y*) there is evidence of discriminant validity (Marôco, 2014).

The reliability of the UWES scores was investigated using internal consistency estimates, Composite Reliability (CR), the ordinal Cronbach's alpha coefficient (α) and the ordinal omega coefficient (ω) for each factor and for the total scale. The second-order factor reliability was also calculated with the omega coefficient (semTools Contributors, 2016).

The measurement invariance corresponds to the situation where links between the items and the construct studied are invariant for different contexts, such as countries, religions, times, or languages (Gregorich, 2006; Little, 2013; Brown, 2015; Hayduk, 2016). This should be tested on the construction or adaptation plan of any psychometric instrument (Edwards et al., 2017). This analysis will show if in different measurement conditions we are measuring the same attributes. Without evidence for this, the basis for drawing inferences will be severely compromised, and, as a result, the findings of possible differences between samples of different groups cannot be unambiguously interpreted (Horn and McArdle, 1992).

Establishing measurement invariance consists of the estimation of an increasingly constrained set of structural equation models, and then evaluating if the differences between those models are significant (van de Schoot et al., 2012). To detect whether the same three-factor model holds in each group of professionals, a group of nested models with indications of equivalence is needed (Marôco, 2014). For the three-factor first-order version of the UWES-9, the first model to be tested is one where only the factor loadings are equal across groups, but the thresholds are allowed to differ between groups; this is called metric invariance and assesses if the professionals in the different occupational groups attribute the same meaning to the three work engagement factors. Next, to assess scalar invariance, a model in which the thresholds and loadings are forced to be the same is tested; it indicates that the three work engagement latent variables (in other words, the loadings), and that the levels of the thresholds are equal for the three different kinds of professionals (van de Schoot et al., 2012). As a result, the occupational groups can be compared on their scores for the vigor, absorption and dedications variables. The next step tests a model in which the residual variances are also forced to be equal across the three groups of professionals, achieving the so-called full uniqueness Measurement Invariance; if obtained, the explained variance for all items is the same for the occupational groups. This means the three factors are measured identically across professional groups. Finally, a model in which the latent means are the same for the different groups is tested. This approach is based on the recommendations of Millsap and Yun-Tein (2004), who specifically discuss the measurement invariance for ordered-categorical measures using the theta-parameterization. Most researchers still use multi-group confirmatory maximum likelihood factor analysis of a Person covariance matrix irrespective of the nature of the measures (Koh and Zumbo, 2008). The approach used in this study tries to be more precise and adequate given the kind of measures adopted (Hirschfeld and von Brachel, 2014).

For the second-order model, a different approach (with more models) is needed. A group of seven models were tested, based on the recommendations of Chen et al. (2005) to test the invariance in second-order models, and in the recommendations of Millsap and Yun-Tein (2004) due to the ordered-categorical nature of our measures. The following models with increasing constraints were compared: (a) configural invariance, (b) first-order factor loadings, (c) second-order factor loadings, (d) thresholds of measured variables, (e) intercepts of first-order factors, (f) disturbances of first-order factors, and (g) residual variances of observed variables. Invariance is supported if the ΔCFI < 0.01 between constrained and free models is presented (Cheung and Rensvold, 2002) and if Δχ^2^ test comparing the fit of the constrained vs. free models is not statistically significant (Satorra and Bentler, 2001).

All statistical analyses were performed with R (R Core Team, 2017) and Rstudio (RStudio Team, 2017). The descriptive statistics were obtained with the skimr package (Arino de la Rubia et al., 2017). The CFA analysis was conducted with the lavaan package (Rosseel, 2012) using the Weighted Least Squares Means and Variances (WLSMV) estimation method. The reliability estimates and measurement invariance (for the first-order model) were calculated with the semTools package (semTools Contributors, 2016), the measurement invariance for the second-order model was analyzed with lavaan (Rosseel, 2012), the Mardia Kurtosis (Mardia, 1970) was assessed with the psych package (Revelle, 2017), and the doBy package (Højsgaard and Halekoh, 2016) for the percentiles. The comparisons of the raw levels of the factors (estimated as the mean of the items for each factor) between the occupational groups were tested using one-way ANOVA with pairwise comparisons using the stats package (R Core Team, 2017). The effect sizes were calculated using Partial Eta Squared from the lsr package (Navarro, 2015).

## Results

Results are presented following the *Standards for Educational and Psychological Testing* framework.

### Internal structure validity evidence

#### Dimensionality

##### Distributional properties of items

As shown in Table 3 (UWES-17) and Table 4 (UWES-9), there were no items with skewness and kurtosis values that were indicative of severe normality deviations (Kline, 2016), the Mardia's Multivariate Kurtosis for the UWES-17 items was 279.4, *p* < 0.001, and for the UWES-9 version it was 189.4, *p* < 0.001. No outliers were deleted. All possible answer values for each item were also present. The mean for most items was close to 4. Therefore, factor analysis with WLSMV, to account for the ordinal level of measurement of the items, can be done without concerns about the validity of its estimates.

**Table 3:** UWES-17 descriptive statistics (*n* = 3459).

**Item**	**Missing %**	***Mean***	***SD***	**Min**	**Max**	**Skewness**	**Kurtosis**	**Histogram**
UWES1^V^	2.40	4.66	1.36	0	6	−1.27	1.15	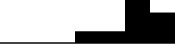
UWES2^D^	2.52	5.34	1.13	0	6	−2.12	4.57	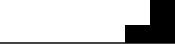
UWES3^A^	2.86	4.74	1.32	0	6	−1.53	2.30	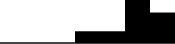
UWES4^V^	2.60	4.74	1.28	0	6	−1.31	1.50	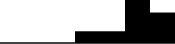
UWES5^D^	2.95	4.62	1.44	0	6	−1.19	0.83	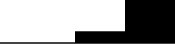
UWES6^A^	2.52	4.04	1.78	0	6	−0.93	−0.17	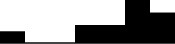
UWES7^D^	2.89	4.41	1.55	0	6	−0.99	0.22	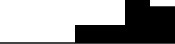
UWES8^V^	2.72	4.10	1.73	0	6	−0.88	−0.25	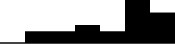
UWES9^A^	2.72	4.36	1.62	0	6	−1.02	0.22	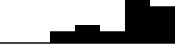
UWES10^D^	2.75	4.98	1.33	0	6	−1.52	1.91	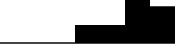
UWES11^A^	3.70	4.26	1.56	0	6	−0.94	0.17	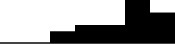
UWES12^V^	2.80	4.22	1.66	0	6	−0.92	−0.08	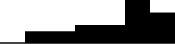
UWES13^D^	3.15	4.65	1.50	0	6	−1.17	0.70	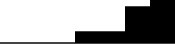
UWES14^A^	3.24	4.06	1.76	0	6	−0.87	−0.24	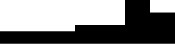
UWES15^V^	2.54	4.65	1.40	0	6	−1.18	0.90	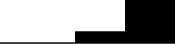
UWES16^A^	2.78	3.78	1.88	0	6	−0.56	−0.86	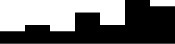
UWES17^V^	2.46	4.84	1.35	0	6	−1.36	1.44	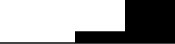

*V, Vigor items; D, Dedication items; A, Absorption items*.

**Table 4:** UWES-9 descriptive statistics (*N* = 3887).

**Item**	**Missing %**	**Mean**	***SD***	**min**	**max**	**Skewness**	**Kurtosis**	**Histogram**
UWES1^V(1)^	3.01	4.65	1.35	0	6	−1.27	1.13	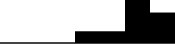
UWES4^V(2)^	3.09	4.72	1.28	0	6	−1.31	1.44	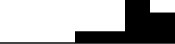
UWES5^D(3)^	3.37	4.58	1.45	0	6	−1.17	0.77	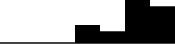
UWES7^D(4)^	3.34	4.38	1.56	0	6	−0.99	0.19	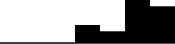
UWES8^V(5)^	3.16	4.08	1.74	0	6	−0.88	−0.27	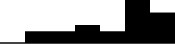
UWES9^A(6)^	3.16	4.32	1.64	0	6	−1.01	0.16	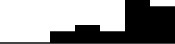
UWES10^D(7)^	3.19	4.96	1.35	0	6	−1.52	1.91	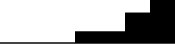
UWES11^A(8)^	4.12	4.28	1.56	0	6	−0.98	0.25	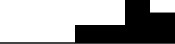
UWES14^A(9)^	3.73	4.06	1.77	0	6	−0.87	−0.27	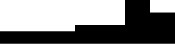

*V, Vigor; D, Dedication; A, Absorption; (#), Item number in the UWES-9 version*.

##### Factor related validity evidence

The three-factor hypothesized model of UWES-17 demonstrated acceptable fit to the data ($\chi_{\left(116\right)}^{2}$ = 3,166.950, *p* < 0.001, *n* = 3,196; *CFI* = 0.994; *TLI* = 0.993; *NFI* = 0.993; *RMSEA* = 0.091; *P*(*rmsea* ≤ 0.05) < 0.001, IC90 ]0.088; 0.093[), since *CFI, TLI*, and *NFI* values were above 0.95, but RMSEA values were above 0.08 (Marôco, 2014), while the factor loadings of all items were above 0.57.

**Figure 1:**
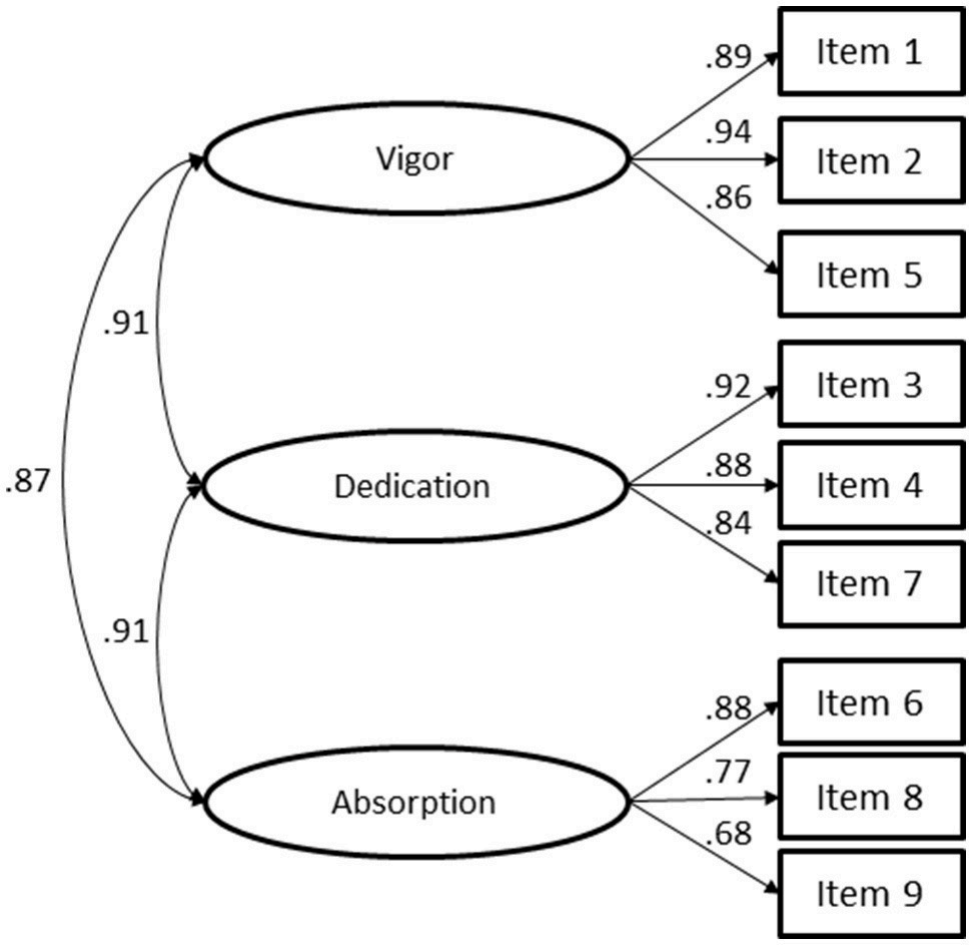
Confirmatory Factor Analysis of the Utrecht Work Engagement Scale (9 items) with Portuguese rescue workers ($\chi_{\left(24\right)}^{2}$ = 981.892, *p* < 0.001, *N* = 3,623; *CFI* = 0.997; *TLI* = 0.995; *NFI* = 0.996; *RMSEA* = 0.105; *P*(rmsea ≤ 0.05) < 0.001, IC90 ]0.099; 0.111[).

For the UWES-9 hypothesized model, the fit was considered acceptable (Figure 1; $\chi_{\left(24\right)}^{2}$ = 981.892, *p* < 0.001, *n* = 3,623; *CFI* = 0.997; *TLI* = 0.995; *NFI* = 0.996; *RMSEA* = 0.105; *P*(rmsea ≤ 0.05) < 0.001, IC90 ]0.099; 0.111[) with *CFI, TLI*, and *NFI* values above 0.95, and RMSEA values above 0.08. The factor loadings of all items were above 0.68. As the UWES-9 showed a better fit to the sample data, we proceeded with the analysis of this version of the UWES.

##### Convergent validity evidence

For the UWES-17, AVE was satisfactory for all dimensions: vigor = 0.65, dedication = 0.72, and absorption = 0.52. For the nine-item version, UWES-9, AVE was good for all dimensions, vigor = 0.80, dedication = 0.78, and absorption = 0.61.

##### Discriminant validity evidence

The discriminant validity was assessed by comparing the AVE of the factors with the squared correlation of the factors, as proposed by Fornell and Larcker (1981) and described in Marôco (2014). Discriminant validity evidence is obtained when the AVE for two scales is larger than the squared Pearson correlation between the two scales. For the UWES-17, AVE_vigor_ = 0.65 and AVE_dedication_ = 0.72 were smaller than ${\text{r}}_{\text{VD}}^{2}$ = 0.86, AVE_absorption_ = 0.52 and AVE_dedication_ = 0.72 were smaller than ${\text{r}}_{\text{AD}}^{2}$ = 0.85 and AVE_vigor_ = 0.65 and AVE_absorption_ = 0.52 were smaller than ${\text{r}}_{\text{VA}}^{2}$ = 0.86. For the UWES-9, AVE _vigor_ = 0.80 and AVE_dedication_ = 0.78 were not larger than ${\text{r}}_{\text{VD}}^{2}$ = 0.83, AVE_absorption_ = 0.61 and AVE_dedication_ = 0.78 were smaller than ${\text{r}}_{\text{AD}}^{2}$ = 0.82, and AVE_vigor_ = 0.80 and the AVE_absorption_ = 0.61 are, respectively, larger and smaller than ${\text{r}}_{\text{VA}}^{2}$ = 0.76.

##### Second-order construct

**Figure 2:**
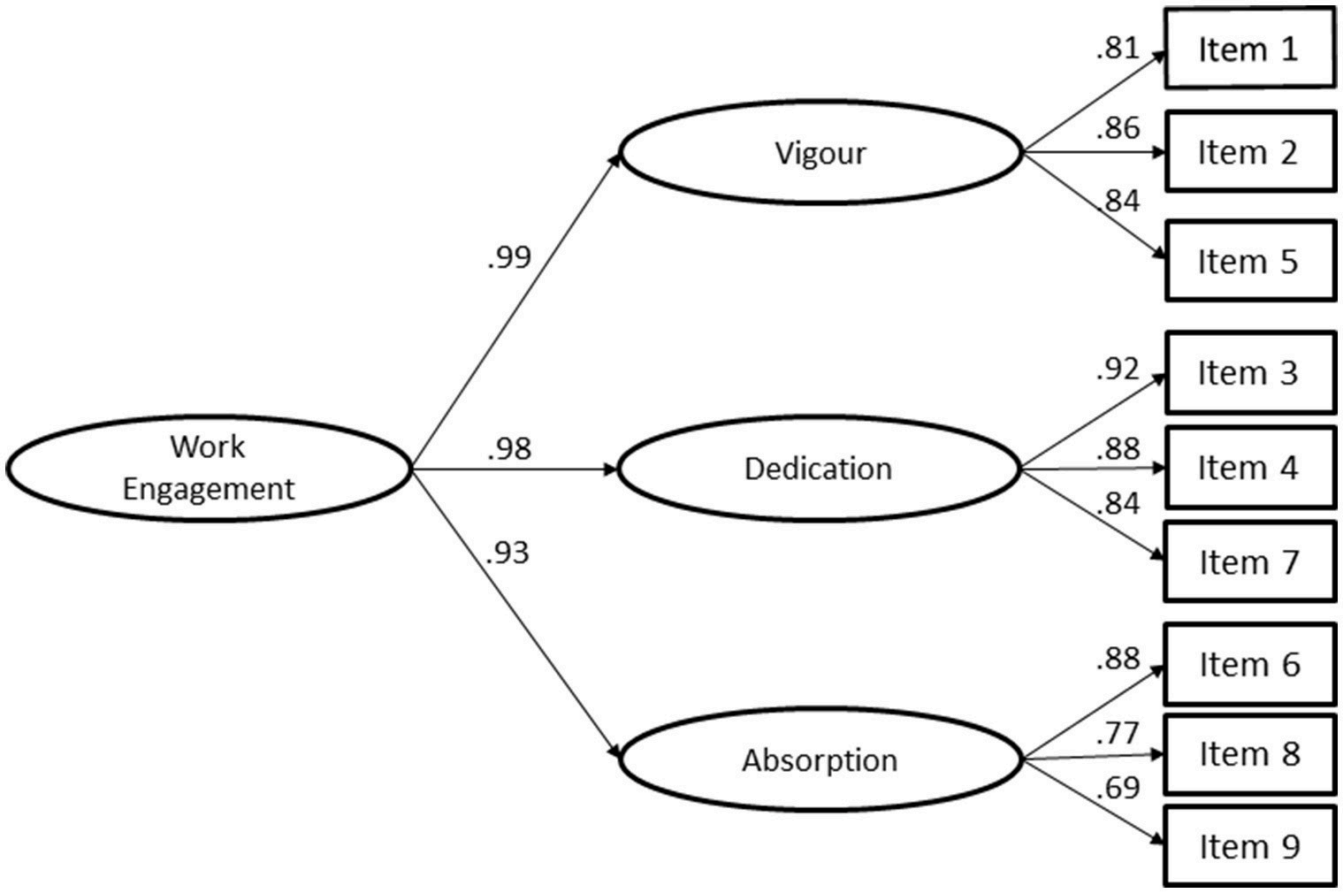
Confirmatory Factor Analysis of the Utrecht Work Engagement Scale (9 items) second-order latent factor with Portuguese rescue workers ($\chi_{\left(24\right)}^{2}$ = 498.849, *p* < 0.001, *n* = 3,623; *CFI* = 0.998; *TLI* = 0.997; *NFI* = 0.998; *RMSEA* = 0.074; *P*(rmsea ≤ 0.05) < 0.001, IC90 ]0.068; 0.080[).

When there is a higher order latent variable (work engagement) that is hypothesized to consider the relations among the lower order factors (vigor, dedication, and absorption in this case), a second-order latent model is admissible (Chen et al., 2005). Another possibility to consider second-order latent models is when the first-order dimensions are substantially correlated with each other (Marôco, 2014), which is verified in the first-order three-factor UWES-9 model. For the UWES-9 model, a second-order latent factor was proposed. A correlation between the residual variances of item 1 and item 4 of the Vigor dimension was added. Regarding the UWES-9 with a second-order latent factor, the fit was very good (Figure 2; $\chi_{\left(24\right)}^{2}$ = 498.849, *p* < 0.001, *n* = 3,623; *CFI* = 0.998; *TLI* = 0.997; *NFI* = 0.998; *RMSEA* = 0.074; *P*(rmsea ≤ 0.05) < 0.001; IC90 ]0.068; 0.080[).

#### Reliability of the scores: internal consistency

To assess the internal consistency, first we used the Composite Reliability of the factors. Also standard Cronbach's alpha coefficient (α) and omega coefficient (ω) were used for each dimension proposed in the UWES-17 and in the UWES-9, and to assess the consistency of the total scales (Table 5).

**Table 5:** Internal consistency of UWES dimensions.

**UWES tri-factor dimensions**	**UWES-17**			**UWES-9**		
	**α_Total sample_**	**ω_Total sample_**	***CR*_Total sample_**	**α_Total sample_**	**ω_Total sample_**	***CR*_Total sample_**
Vigor	0.90	0.89	0.94	0.90	0.89	0.94
Dedication	0.93	0.90	0.96	0.91	0.89	0.93
Absorption	0.86	0.84	0.93	0.82	0.79	0.86
Total	0.96	0.95	–	0.95	0.94	–

Reliability of the higher-order construct was very good. The proportion of the second-order factor explaining the total score (ω_L1_) was 0.93, the proportion of the second-order factor explaining the variance of the first-order factor level (ω_L2_) was 0.98, and the proportion of observed variance explained by the second-order factor after controlling for the uniqueness from the first-order factor (ω_partial L1_) was 0.94. The structural weights for the second-order factor model were: vigor (β = 0.99; *p* < 0.001); dedication (β = 0.98; *p* < 0.001); and absorption (β = 0.93; *p* < 0.001).

#### Measurement invariance

To detect whether the same original three-factor (first-order model) holds in each occupation (Table 6), a group of nested models with indications of equivalence is needed (Marôco, 2014). The hypothesized scale invariance was supported by the Cheung and Rensvold (2002) ΔCFI criterion, but not by the Δχ^2^ criterion (Satorra and Bentler, 2001), although the second criterion is more restrictive. According to the Cheung and Rensvold (2002) criterion, the results supported the full uniqueness measurement invariance between the occupational groups (nurses, firefighters, and police officers). However, there were significant differences between the means of the UWES dimensions for the different occupational groups. It could be noticed that these comparisons are valid since the measures showed scalar invariance.

**Table 6:** UWES-9 first-order three-factor latent model comparison between occupational groups of rescue workers.

**Model**	**χ^2^**	***df***	**χ^2^/*df***	***CFI* scaled**	**Δχ^2^**	**Δ*CFI* scaled**
Configural	1,126	72	15.64	0.963	–	–
Metric	1,152	84	13.71	0.971	25^*^	0.008
Scalar	1,571	168	9.35	0.973	479^***^	0.002
Full uniqueness	2,242	186	12.05	0.964	512^***^	0.009
Latent means	8,440	192	43.96	0.920	332^***^	0.043

* p < 0.05;

*** *p < 0.001*.

The test of the measurement invariance for the second-order model for categorical variables is shown in Table 7. According to ΔCFI criteria, only partial (metric) second-order invariance is supported in the second-order UWES-9 model for the three different occupational groups.

**Table 7:** UWES-9 second-order three-factor latent model comparison between occupational groups of rescue workers.

**Model**	**χ^2^**	***df***	**χ^2^/*df***	***CFI* scaled**	**Δχ^2^**	**Δ*CFI* scaled**
Configural	609	73	8.34	0.979	–	–
First-order loadings invariance	638	85	7.51	0.982	7.60^*^	0.003
Second-order loadings invariance	647	89	7.27	0.984	1.86^*ns*^	0.002
Thresholds of measured variables	1,098	173	6.35	0.982	66.00^***^	0.002
Intercepts of first-order factors invariance	7,533	179	42.08	0.920	187.00^***^	0.062
Disturbances of first-order factors invariance	7,909	184	42.98	0.917	116.00^***^	0.003
Residual variances of observed variables invariance	8,378	202	41.48	0.929	44.90^***^	0.012

ns p > 0.05;

* p ≤ 0.05;

*** *p < 0.001*.

### Dimension comparisons

Considering rescue workers' occupations, Table 8 presents percentiles and mean levels of each work engagement dimension, and a comparison between nurses, firefighters, and police officers. For the hypothesized differences between occupational groups, some statistical significant differences in work engagement were found between the three groups (Table 9), being highest among firefighters and lowest among nurses. Percentile analyses reveal the exact value for each category and occupation, reflecting the tendency revealed by the mean analysis of each sample.

**Table 8:** Comparative analysis between rescue workers (means, standard deviations, and percentiles).

**UWES-9 dimension**	**Nurses (*n* = 1494)**		**Firefighters (*n* = 1299)**		**Police officers (*n* = 403)**		***F***	***df***	***p***	**$\eta_{\text{p}}^{2}$**	**Nurses**			**Firefighters**			**Police officers**		
	***M***	***SD***	***M***	***SD***	***M***	***SD***					**25**	**50**	**75**	**25**	**50**	**75**	**25**	**50**	**75**
Vigor	4.11	1.29	4.91	1.18	4.74	1.12	181.8	2 3,735	<0.001	0.09	3.00	4.33	5.00	4.67	5.00	5.67	4.33	5.00	5.67
Dedication	4.27	1.29	5.10	1.18	4.78	1.24	181.0	2 3,714	<0.001	0.09	3.33	4.67	5.33	5.00	5.33	6.00	4.33	5.00	5.67
Absorption	3.85	1.36	4.73	1.27	4.21	1.26	181.1	2 3,688	<0.001	0.09	3.00	4.00	5.00	4.00	5.00	5.67	3.33	4.67	5.00

**Table 9:** Pairwise comparisons (*t*-test with pooled *SD*).

	**Vigor** ***p-*****values**		**Dedication** ***p-*****values**		**Absorption** ***p-*****values**	
	**Nurses**	**Firefighters**	**Nurses**	**Firefighters**	**Nurses**	**Firefighters**
Firefighters	<0.001	–	<0.001	–	<0.001	–
Police officers	<0.001	0.015	<0.001	<0.001	<0.001	<0.001

## Discussion

### Hypotheses findings

Considering the psychometric properties of data gathered with the UWES in the present sample, our hypotheses were confirmed since UWES-17 and UWES-9 presented validity evidence that supports its usage among rescue workers (respectively H1 and H2), and UWES-9 presented measurement invariance that allows for comparing occupational groups (H3). In fact, the data presented better psychometric properties for the UWES-9 and supported a second-order latent factor for the three-factor structure due to strong correlations between the first-order dimensions. This solution is supported by other international studies, presenting better goodness-of-fit indexes for the shorter version of the instrument (Nerstad et al., 2010; Hernandez-Vargas et al., 2016). The convergent validity evidence and reliability of the scores were good, although the discriminant validity was not acceptable, leading to the proposal of a second-order latent factor. There have been several reports that discuss the dimensionality of work engagement as assessed by UWES (see Table 1). Our data suggests that work engagement can be defined as a second-order factor that is reflected in the workers' vigor, dedication, and absorption. Thus, we suggest that work engagement can be estimated as a single value in research contexts and in clinical settings facilitating the use of the UWES. A second-order latent factor has advantages over a single first-order factor for the UWES as proposed by several researchers (see Table 1) since it allows for the estimation of a single value for work engagement while retaining the three first-order factors proposed (Schaufeli and Bakker, 2003).

Regarding H4 (UWES-9 can evaluate different occupational groups of rescue workers), and considering rescue workers' occupation specificities, work engagement levels varied; despite being moderate to high, they were higher among firefighters and lower among nurses. This can be explained by the fact that Portuguese firefighters are traditionally volunteers (paid or not), being highly engaged in their tasks and frequently assuming physical risks to help in rescue situations (e.g., the tragedy of the Portuguese forest fire during June 2017 at Pedrógão Grande where a firefighter died, and several were severely burned; or the Portuguese forest fire during the summer of 2013 at Caramulo where eight firefighters died). According to Portuguese data on firefighters, in 2014 there were 42,000 firefighters, only 6,000 of whom were professionals and the remaining were volunteers, despite all of them being trained by the Portuguese Firefighters' National School. Moreover, some studies explain that aid workers, such as firefighters, can present a sense of coherence, a concept developed by Antonovsky (1987). Related to the salutogenic theory, it is defined as the “*ability of the persons to understand what happens around them, to what extent they were able to manage the situation on their own or through significant others in their social network, and the ability to find meaning in the situation*” (Eriksson and Lindström, 2005, p. 460). This can help someone to better cope with traumatic events life adversities (Veronese and Pepe, 2014; Veronese et al., 2016). The study of 464 firefighters by Dudek and Koniarek (2000) revealed that post-traumatic stress symptoms were associated with a low level of sense of coherence, especially if they already have post-traumatic stress disorder. Additionally, firefighters, like police officers, share some personality traits like sensation seeking (Perrott and Blenkarn, 2015), that increase their motivation to pursue certain occupations. Nurses presented the lowest work engagement values, maybe due to the external demands their occupation is facing, namely lack of human or material resources, conflicts with patients or being aggressed by them or their families, etc. (Lin et al., 2016; Marôco et al., 2016). Indeed, recent austerity measures adopted in countries such as Portugal have had significant negative effects on the availability of healthcare system resources, including reduced staffing and employment conditions (Dussault and Buchan, 2014). Regarding the work engagement dimensions, it is important to notice that they are differentiated according to each rescue occupation, since vigor is related to physical energy, used more by firefighters and police officers, while dedication is clearly higher among firefighters who voluntarily seek this activity. Finally, absorption must be interpreted according to each task, which was higher among firefighters and police officers, especially when they are in rescue operations where time “flies,” while nurses have more rigid protocols to obey, accomplishing schedules and some rigid tasks. Results are similar to other studies where rescue workers present moderate to high work engagement levels (see Table 1), with nurses having lower work engagement than police officers (Schaufeli et al., 2006), and a lack of studies about firefighter's work engagement (curiously the one found was with a Portuguese sample).

### Limitations and recommendations for future research

This study has some limitations, namely having a convenience sample collected using participants' voluntary participation. In the future, respecting voluntary participation, we would like to attract more participants, trying to have equal representation among the occupations. In fact, in this sample the percentages were not homogeneous, with nurses representing half of the sample, and police officers comprising only 11% of the sample. Firefighters were also included, but not other rescuers such as medical emergency staff, civil protection technicians, social workers or psychologists, who, for example, during the summer of 2017 in Portugal, participated in rescue tasks for the population due to the tragic forest fires. Furthermore, we did not have any source of evidence-based on relations to other variables, which can provide external validity evidence of UWES when compared with other concurrent or divergent measures (American Educational Research Association et al., 2014). This was a consequence of collecting data from each occupational group separately or during a different year, according to different research projects developed in the same University research unit, which were addressing different topics, having in common the measurement of work engagement with UWES. In the future, we would like to increase the sample, with replication efforts to collect data among more diverse rescue occupations. Also, we intend to have more homogeneity between rescue workers' samples sizes, having thus a stronger national representativeness, which can be obtained using web-based versions (Ilieva et al., 2002) of UWES (with other measures) through, for example, LimeSurvey (Limesurvey GmbH, 2017). The advantages of web-based surveys can also include increased delivery speed, control and monitoring, automatic data validation, and decreased data entry error (Falletta and Combs, 2002). Finally, we would like to combine work engagement measurement with other variables such as burnout, trauma or job satisfaction, as frequently studied in numerous research (e.g., Argentero and Setti, 2011; Ângelo and Chambel, 2015; Espinoza-Parra et al., 2015).

### Theoretical implications

The study has some theoretical implications, enabling a better understanding of work engagement dimensions related with each occupational group specificities (e.g., vigor is higher among firefighters and police officers who use more physical energy). Additionally, the recent Schaufeli (2017a) publication of the general engagement scale (UGES), with 9 items evaluating daily activities and not only work/job activities will broad the evaluation of engagement and its relationship with psychological variables such as positive and negative affect, satisfaction with life, personality traits (Schaufeli, 2017b), or sense of coherence and trauma (Dudek and Koniarek, 2000; Veronese and Pepe, 2017). Thus, work engagement seems to be an important concept to study among workers and organizations, considering that organizations are facing more competition, innovation, and constant changes, and also a decrease of resources (Graffigna, 2017). Engaged workers are thus an important element for organizations' productivity or to the care/services they provide to citizens.

### Practical implications

Regarding practical implications, this validation of a Portuguese version of UWES can contribute to the improvement of rescue workers' quality of life, since these results can alert individuals and institutions about the need to explore work engagement level to buffer against negative physiological health effects (Lenthall et al., 2009; Kumar, 2011; Ray et al., 2013; Setti and Argentero, 2014; Burton et al., 2017). Moreover, reference values for the Portuguese firefighters, nurses and police officers' populations make it possible to establish comparisons with other occupational groups of rescue workers (e.g., physicians, social workers). Taken together, the findings of the present study have important ecological implications for Portuguese policymakers and human resource managers who can structure rescue workers' work conditions, or develop intervention programs to promote their work engagement, and therefore help prevent burnout and other mental health conditions that affect these professionals' well-being.

## Conclusions

Regarding the four research hypotheses, all were confirmed, since convergent validity evidence was found. Due to the lack of discriminant validity evidence, a second-order latent factor was proposed. Additionally, reliability evidence presented good values, and we tested measurement invariance for ordinal variables, using an innovative approach for categorical variables. Moreover, UWES can discriminate work engagement levels for each occupational group of rescue workers. Thus, this UWES version can be used to collect more data for future comparative studies. Additionally, due to natural or human causes (e.g., forest fires, multi-victim accidents, terrorist attacks), rescue workers play an increasingly important role in modern society, and the work engagement concept will help to better understand rescue workers' motivation to continue performing their tasks even when they risk their own life and frequently face tragedies.

## Ethics statement

This study was carried out in accordance with the recommendations of guidelines of the Faculty of Psychology and Education Sciences of the University of Porto with written informed consent from all subjects. All subjects gave written informed consent in accordance with the Declaration of Helsinki. The protocol was approved by the Ethics Committee of the Faculty of Psychology and Education Sciences of the University of Porto.

## Author contributions

All authors of this research paper have directly participated in the planning, execution, or analysis of this study. More specifically, JS wrote the paper, and with JM performed all statistical analysis and its discussion. JS and AM-P discussed engagement concept, theoretical framework, and its results interpretation, JS and CQ discussed rescue workers' engagement, theoretical framework and engagement results.

### Conflict of interest statement

The authors declare that the research was conducted in the absence of any commercial or financial relationships that could be construed as a potential conflict of interest.
